# Supplementary material for: New Eocene primate from Myanmar shares dental characters with African Eocene crown anthropoids
Source: Nat Commun. 2019 Aug 6;10:3531. doi: 10.1038/s41467-019-11295-6 (PMC6684601; doi:10.1038/s41467-019-11295-6)
Supplement: Supplementary file 1 — Supplementary Information [file 41467_2019_11295_MOESM1_ESM.pdf]

Supplementary Information

New Eocene primate from Myanmar shares dental characters with African Eocene crown anthropoids

Jaeger et al.

Contents

Supplementary Figure 1 (additional illustrations of *Aseanpithecus myanmarensis*)**…………….p. 2**

Supplementary Note 1: bodyweight estimation of *Aseanpithecus*……………………………..p. 3

Supplementary Note 2: estimation of orbit diameter of *Aseanpithecus* (with suppl. figs 2-3)**..p. 3**

Supplementary Note 3: phylogenetic analysis (with Supplementary Figs 4-7)**……..…………p. 6**

Selected taxa and dataset……………………………………………………………………p. 6

Analyses and results…………………………………………………………………………p. 7

List of characters ……………………………………………………………………………p. 17

Supplementary References……………………………………………………………………p. 30

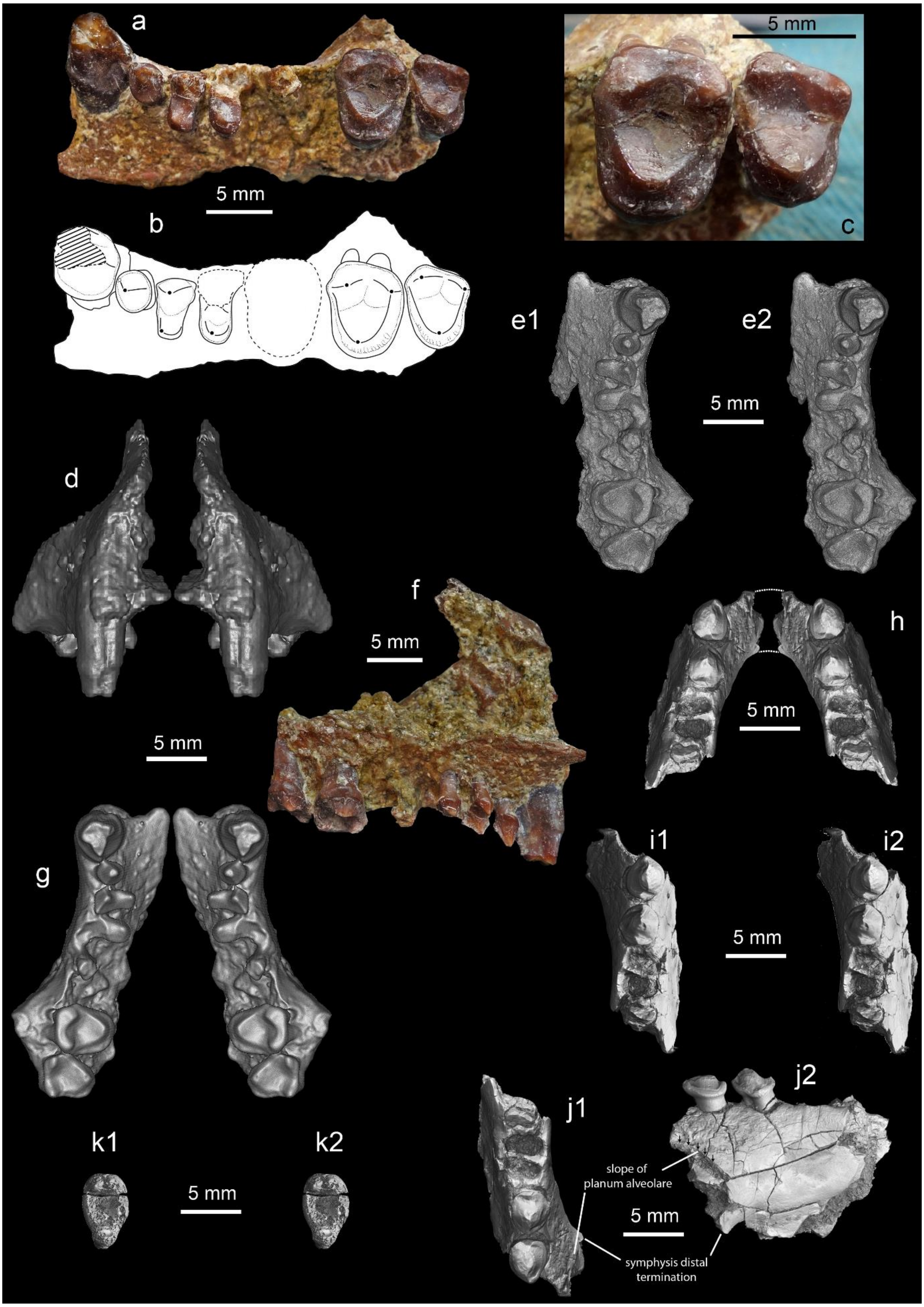

Supplementary Figure 1. *Aseanpithecus myanmarensis*. **a-g**, holotype maxilla with C-M^3 NMMP 93; **a**, photograph in occlusal view; **b**, interpretive drawing with reconstructed P^4 and M^1 outlines; **c**,

photograph of M^2-M^3 in occlusal view; **d**, symmetrized maxilla in anterior view; **e1-e2**, stereo-pair in occlusal view (3D rendering images); **f**, photograph in medial view; **g**, symmetrized maxilla in occlusal view; **h-j**, CT-scan images of NMMP 95, right mandible fragment with P_2-P_3; **h**, symmetrized mandible in occlusal view; **i1-i2**, stereo-pair in occlusal view; **j1-j2**, annotated symphyseal region of NMMP 95 showing the preserved parts of planum alveolare and possible distal termination of the symphysis; **k1-k2**, stereo-pair of the right M_3 NMMP 96 in occlusal view (CT-scan images). Drawing 1b by Sabine Riffaut.

Supplementary Note 1: bodyweight estimation of *Aseanpithecus*

We have estimated the bodyweight of *Aseanpithecus* based on the holotype maxilla NMMP 93, the mandible NMMP 95 preserving no molar and NMMP 96 being a M_3, a tooth considered as a rather imprecise bodyweight estimator[1].

The M^1 being absent on the maxilla, we have estimated the bodyweight of NMMP 93 with the surface area of M^2 (=length x breadth) using the regression of ref.[1] for primates: $\ln B = 1.37 * \ln S + 3.49$ where B is the bodyweight and S the surface area of M^2. 95% confidence intervals having also being calculated using the formula in ref.[1]. We have obtained a bodyweight prediction for NMMP 93 of 3.08 kg with a 95% confidence interval of 2.81-3.38 kg.

Supplementary Note 2: estimation of orbit diameter of *Aseanpithecus*

The diameter of the orbit of NMMP 93 was estimated using the methodology of ref.[2]. This methodology is using 3 points of the orbit plane (X, Y, Z) to calculate the orbit radius. The original methodology for point selection of ref.[2] is using the inferiormost point of the orbit (Y) and two other points along the orbit (X and Z) equally distant from Y. The preserved portion of the orbit accessible on NMMP 93 do not allowing such a positioning of the points, we have placed X and Z at the two extremities of the preserved rim to maximize the length of orbit rim used. Y was placed at equal distance from X and Z (Supplementary Fig. 2). NMMP 93 has a total length of preserved rim of 15 mm which is sufficient to obtain a reasonably good estimate of the orbit diameter but not an optimal one[2]. The specimen was oriented following the orbital plane, photographed, and the WY and WZ distances have been subsequently determined with

the software ImageJ. The formula (corrected from that of ref.[2], erroneous) used to calculate the orbit diameter D is: $D = 2OY = \frac{WY^2+WZ^2}{WY}$.

A diameter of 13.4 mm was found for *Aseanpithecus*. Taking an estimated length of 5 mm for the M^1, this orbit diameter of *Aseanpithecus* is proportionally small relative to tooth size, confidently indicating a diurnal activity pattern (Supplementary Fig. 3).

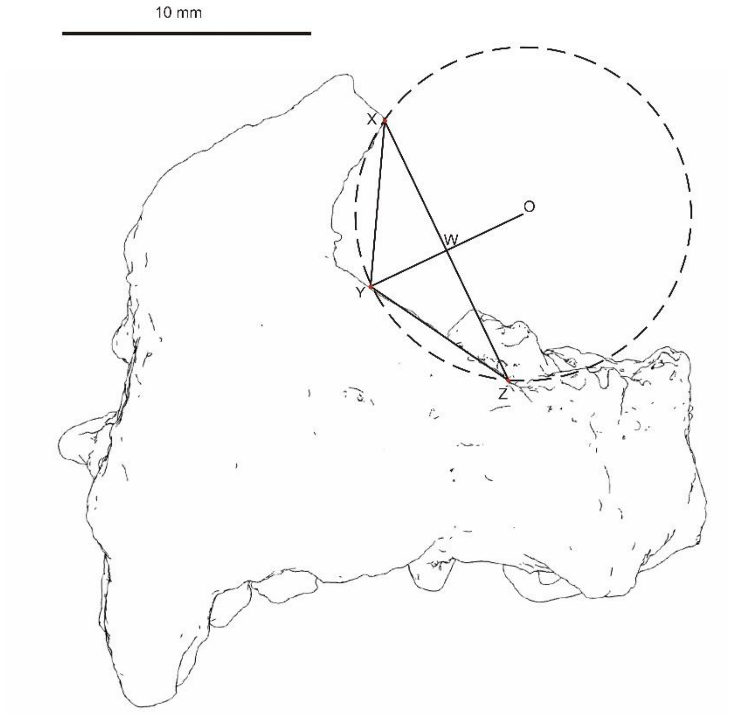

Supplementary Figure 2. Position of the three points (X, Y, Z) placed along the orbit rim to calculate the orbit radius and diameter after orientation of NMMP 93 following the orbit plane. The circle represents the results of the radius/diameter orbit estimation. Owing to the peculiar shape of the preserved orbit rim on NMMP 93, this circle represents only an estimation of the orbit size but is not an attempt of orbit shape reconstruction.

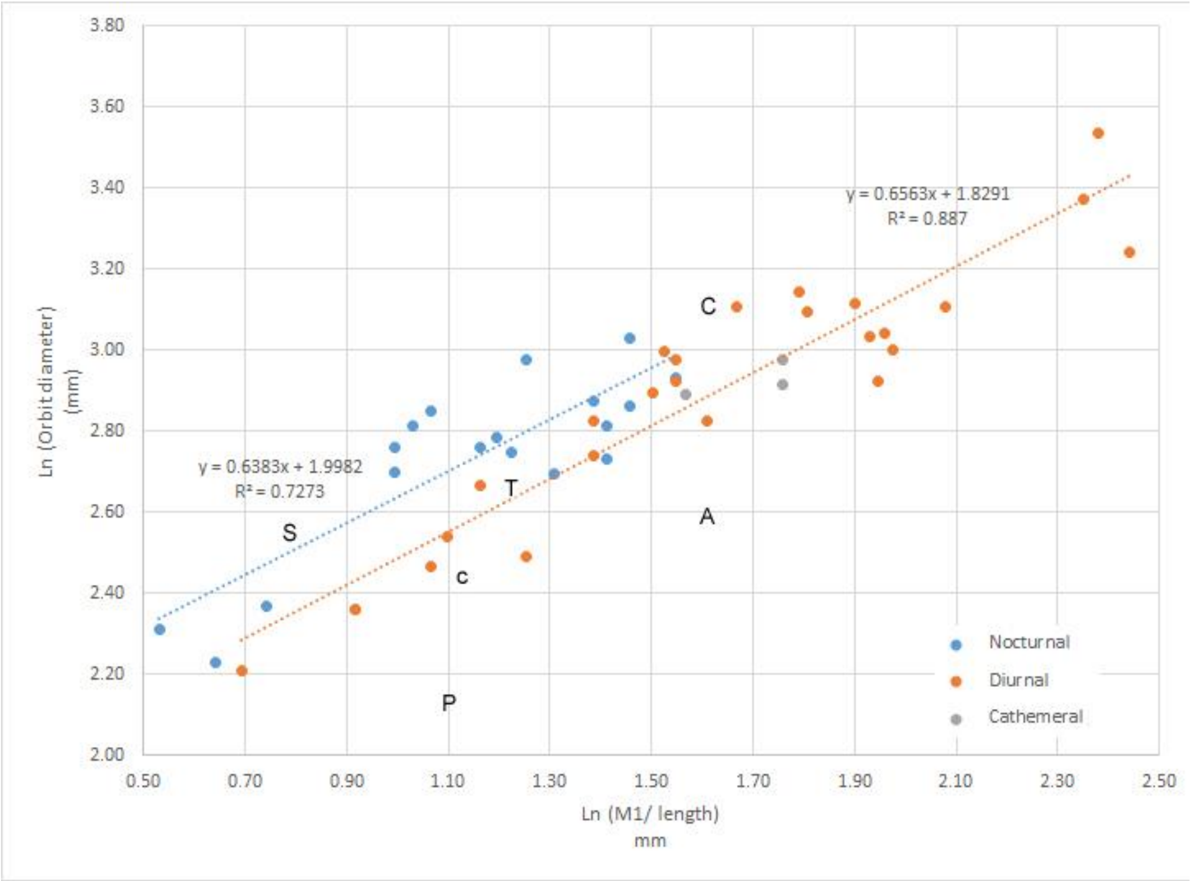

Supplementary Figure 3. Bivariate plot of log-transformed M^1 length versus orbit diameter of 47 extant and 6 fossil primates species. The orbit diameter corresponds to the measurement of the mediolateral diameter of the orbit except for *Aseanpithecus* for which the orbit diameter was estimated. Linear regressions for extant diurnal and nocturnal primates are shown with their equations and coefficients of determination. A M^1 length of 5 mm has been taken for *Aseanpithecus* considering that the M^1 of NMMP 93 was most likely slightly larger than the M^2 which measures 4.71 mm in length. All data except for *Aseanpithecus* from ref[2]. Legend for fossil taxa: A= *Aseanpithecus*, C=*Cantius*, c=*Catopithecus*, P=*Proteopithecus*, S=*Shoshonius*, T=*Tremacebus.*

Supplementary Note 3: phylogenetic analysis

Selected taxa

We have used a dataset with 45 taxa for the phylogenetic analysis. All the taxa are Paleogene fossils with the exception of the extant platyrrhines *Saguinus*, *Saimiri*, *Aotus* and *Callicebus*, the fossil platyrrhine *Dolichocebus* which is dated from the early Miocene[3], and the extant tarsiid *Tarsius*.

Outgroup taxa (5):

Notharctidae: *Donrussellia gallica*+sp., *Cantius eppsi*, *Notharctus robustior*

Adapidae: *Adapis parisiensis*, *Leptadapis magnus*

Ingroup taxa (40):

Omomyidae: *Absarokius abotti*+sp., *Teilhardina americana, Shoshonius cooperi, Hemiacodon gracilis*

Tarsiidae: *Xanthorhysis tabrumi, Tarsius* spp.

Eosimiidae: *Eosimias* spp., *Phenacopithecus* spp., *Bahinia pondaungensis*

Afrotarsiidae: *Afrasia djijidae, Afrotarsius libycus*

Propliopithecidae: *Aegyptopithecus zeuxis, Moeripithecus markgrafi, Propliopithecus chirobates, Propliopithecus* spp.

Oligopithecidae: *Oligopithecus savagei*, *Oligopithecus rogeri*, *Catopithecus browni, Talahpithecus parvus.*

Amphipithecidae: *Myanmarpithecus yarshensis, Pondaungia cotteri* (including *Amphipithecus mogaungensis*), *Siamopithecus eocaenus, Ganlea megacanina, Krabia minuta.*

Platyrrhini: *Dolichocebus gaimanensis*, *Branisella boliviana*, *Saguinus oedipus+nigricollis*, *Saimiri sciureus*, *Aotus trivirgatus+lemurinus*, and *Callicebus moloch.*

Parapithecoidea*: Apidium phiomense, Parapithecus fraasi, Parapithecus grangeri, Qatrania wingi, Biretia* spp.

Proteopithecidae*: Proteopithecus sylviae, Serapia eocaena*.

Family *incertae sedis*: *Aseanpithecus myanmarensis, Bugtipithecus inexpectans, Phileosimias* spp.

Bugtipithecus inexpectans was initially described as an amphipithecid[4] but its status has been recently questioned[6].

Dataset

The present analysis is based on a dataset used in recent publications[5,6] which itself derives from the matrices used in ref[4]. Compared with the one used in refs[5,6], we have newly coded *Propliopithecus* spp.*, Propliopithecus chirobates, Oligopithecus rogeri, Oligopithecus savagei, Talahpithecus parvus, Aseanpithecus myanmarensis, Saguinus oedipus+nigricollis* for the present analysis. *Notharctus robustior, Serapia eocaena, Phileosimias* spp., *Saimiri sciureus, Aotus trivirgatus+lemurinus,* and *Callicebus moloch,* which coding was adapted from ref.[7], were also introduced in the selected taxa.

Analyses

The analyses were performed with PAUP 4b10[8] using a heuristic search (hsearch command) with random step-wise addition (1000 replications with randomized input order of taxa) and tree bisection-reconnection (TBR) branch-swapping options. Characters were equally weighted. We have performed a first set of four analyses without any topological constraint to test the sensibility of the obtained topologies to two parameters:

-the presence or absence of the genus *Phileosimias*, which is already known for being unstable in phylogenies[4]

-the treatment of multiple state characters as partly ordered or fully unordered.

A second set of analyses, topologically-constrained, were performed to test the phylogenetic position of *Aseanpithecus* based on a multisource tree[9] and a recent phylogenetic analysis of anthropoids based on morphological features[10]. We have used for each source a backbone constraint tree reflecting the phylogenetic relationships of the main groups of anthropoids (platyrrhines, propliopithecines, oligopithecines, parapithecoids, amphipithecids, eosimiids) found in these articles.

The phylogenetic relationships of the main groups of haplorhines found in ref.[9] are:

((((((Oligopithecidae, Propliopithecidae), Platyrrhini), Amphipithecidae), (Proteopithecidae, Parapithecoidea)), Eosimiidae), Tarsiidae)

They mainly differ from our previous results by the presence of a Oligopithecidae + Propliopithecidae clade.

Backbone tree 1 used in our analysis based on ref.[9]:

((((((*Oligopithecus savagei*, *Propliopithecus* spp.), *Saguinus* spp.), *Pondaungia cotteri*), (*Proteopithecus sylviae*, *Parapithecus fraasi*)), *Eosimias* spp.), *Tarsius* spp.)

The phylogenetic relationships of the main groups of haplorhines found in ref.[10] are:
((((Parapithecoidea, Propliopithecidae), Platyrrhines), Oligopithecidae), Eosimiidae),

They differ from some of our previous results by the basal position of the Oligopithecidae among advanced anthropoids and from all of our previous results by the position of parapithecoids within the crown-group of anthropoids by the obtention of a Propliopithecidae + Parapithecoidea clade.

Backbone tree 2 used in our analysis based on ref.[10]:
((((*Parapithecus fraasi*, *Propliopithecus* spp.), *Saguinus* spp.), *Oligopithecus savagei*), *Eosimias* spp.),

Results

In all obtained trees, *Aseanpithecus* is reconstructed as an anthropoid. In addition, *Aseanpithecus* is never grouped with basal representatives of the anthropoids (Afrotarsiidae and Eosimiidae) but is nested in every obtained tree within a clade encompassing all derived anthropoids (Amphipithecidae + Parapithecoidea + Proteopithecidae + Oligopithecidae + Propliopithecidae + Platyrrhini). The position of *Aseanpithecus* among this clade of derived anthropoids varies however markedly with taxonomic sampling, treatment of the multistate characters or use of a topological constraint. *Aseanpithecus* is most commonly positioned basally in the clade of derived anthropoids but is also commonly found more deeply nested in this clade than the Proteopithecidae, Parapithecoidea and Oligopithecidae. One of the retrieved topologies even places *Aseanpithecus* as the sister-group of the Oligopithecidae. Because of the apparent instability of *Aseanpithecus* in the obtained trees, reflected by low Bremer and boostrap support values of several internal nodes within the clade of the derived anthropoids, our phylogenetic analysis does not support the attribution of this genus to any known family of anthropoids and rather suggests that it may belong to a new family. We described hereafter the results of each analysis performed.

Analysis 1 (*Phileosimias* retained, some multistate characters treated as ordered) has resulted in 2 equally-parsimonious trees of 1497 steps (see Supplementary Fig. 4A; Consistency index (CI) = 0.3220, Homoplasy index (HI) = 0.6780, CI excluding uninformative characters = 0.3010, HI excluding uninformative characters = 0.6990, Retention index (RI) = 0.5579, Rescaled consistency index (RC) = 0.1796). In the consensus tree, *Aseanpithecus* is nested within a clade of derived anthropoids composed of the Amphipithecidae, a Parapithecoidea+Proteopithecidae clade, the Oligopithecidae, the Propliopithecidae, and the Platyrrhini. *Aseanpithecus* is grouped with *Bugtipithecus* in a clade that is reconstructed as the sister-group of the crown anthropoids, here composed of the platyrrhines, the propliopithecids and the amphipithecids. The most basal representatives of the derived anthropoids on this consensus tree are the Parapithecoidea+Proteopithecidae clade and the oligopithecids.

Analysis 2 (*Phileosimias* retained, all multistate characters treated as unordered) has resulted in 2 equally-parsimonious trees of 1391 steps (see Supplementary Fig. 4B; Consistency index (CI) = 0.3444, Homoplasy index (HI) = 0.6556, CI excluding uninformative characters = 0.3224, HI excluding uninformative characters = 0.6776, Retention index (RI) = 0.5438, Rescaled consistency index (RC) = 0.1873). In the consensus tree, *Aseanpithecus* is again nested within a clade of derived anthropoids composed of the Amphipithecidae, a Parapithecoidea + Proteopithecidae clade, the Oligopithecidae, the Propliopithecidae, and the Platyrrhini. In this analysis, *Aseanpithecus* is the sister-group of a *Bugtipithecus* + Amphipithecidae clade. The *Aseanpithecus* + *Bugtipithecus* + Amphipithecidae clade is sister-group of the crown group of anthropoids, here limited to the platyrrhines and the propliopithecids. The most basal representatives of the derived anthropoids on this consensus tree are the Parapithecoidea+Proteopithecidae clade and the oligopithecids, the latter group being the most basal contrary to the previous analysis.

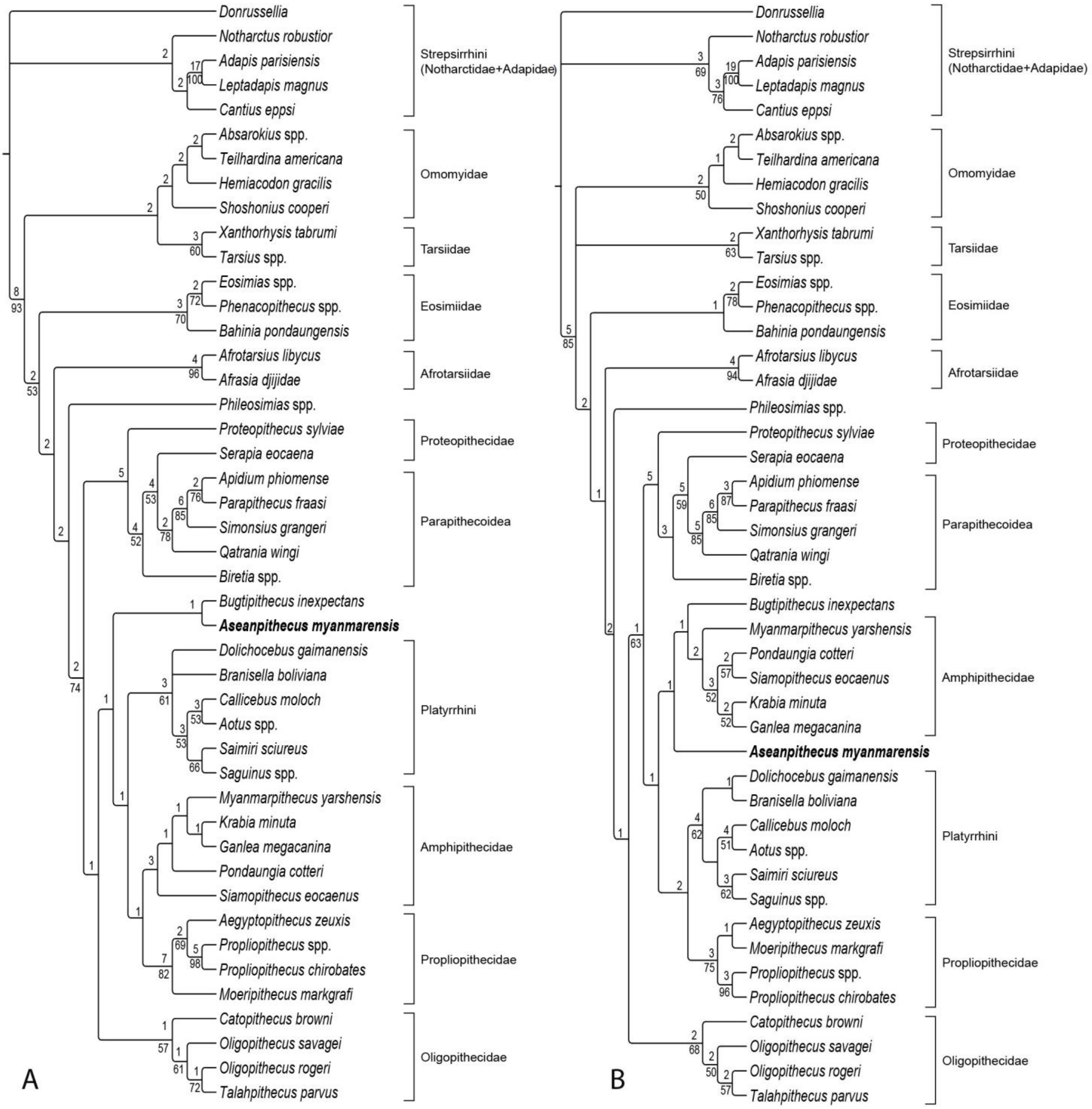

Supplementary Figure 4. A: Strict consensus of two trees of 1497 steps obtained with analysis 1. B: strict consensus of two trees of 1391 steps obtained with analysis 2. Number above branches are Bremer support values. Number below branches are bootstrap support values obtained by a heuristic search with 1000 bootstrap replications (only values >50 are indicated).

Analysis 3 (*Phileosimias* discarded, some multistate characters treated as ordered) has resulted in a single most-parsimonious tree of 1477 steps (see Supplementary Fig. 5A; Consistency index (CI) = 0.3263, Homoplasy index (HI) = 0.6737, CI excluding uninformative characters = 0.3052, HI excluding uninformative characters = 0.6948, Retention index (RI) = 0.5597, Rescaled consistency index (RC) = 0.1827). *Aseanpithecus* is grouped with *Bugtipithecus* and occupies a basal position within a clade of derived anthropoids composed of the Amphipithecidae, a Parapithecoidea+Proteopithecidae clade, the Oligopithecidae, the Propliopithecidae, and the Platyrrhini (same composition as for analyses 1 and 2). On this tree, *Aseanpithecus* is at the same time phylogenetically distant from the primitive Asian anthropoids (Eosimiidae, Afrotarsiidae) and from the Amphipithecidae, which are here nested within the crown-group of anthropoids. The Bremer and boostrap support values are significantly higher in this analysis, especially the clade of derived anthropoids (Bremer support=7, bootstrap support=88%)

Analysis 4 (*Phileosimias* discarded, all multistate characters treated as unordered) has resulted in a single most-parsimonious tree of 1375 steps (see Supplementary Fig. 5B); Consistency index (CI) = 0.3484**,** Homoplasy index (HI) = 0.6516**,** CI excluding uninformative characters = 0.3263**,** HI excluding uninformative characters = 0.6737**,** Retention index (RI) = 0.5443**,** Rescaled consistency index (RC) = 0.1896). As for the three first analyses, *Aseanpithecus* belongs to the clade of derived anthropoids composed of the Amphipithecidae, a Parapithecoidea+Proteopithecidae clade, the Oligopithecidae, the Propliopithecidae, and the Platyrrhini. On the obtained tree, *Aseanpithecus* is more deeply nested with this clade, being reconstructed as the sister group of the Oligopithecidae in a Oligopithecidae + *Aseanpithecus* + *Proteopithecus* + Parapithecoidea clade. The latter clade is sister-group of the crown anthropoids (Platyrrhini+Propliopithecidae). The Amphipithecidae are here the most basal group within the derived anthropoids. Note that the phylogenetic position of tarsiids is not consistent in this tree.

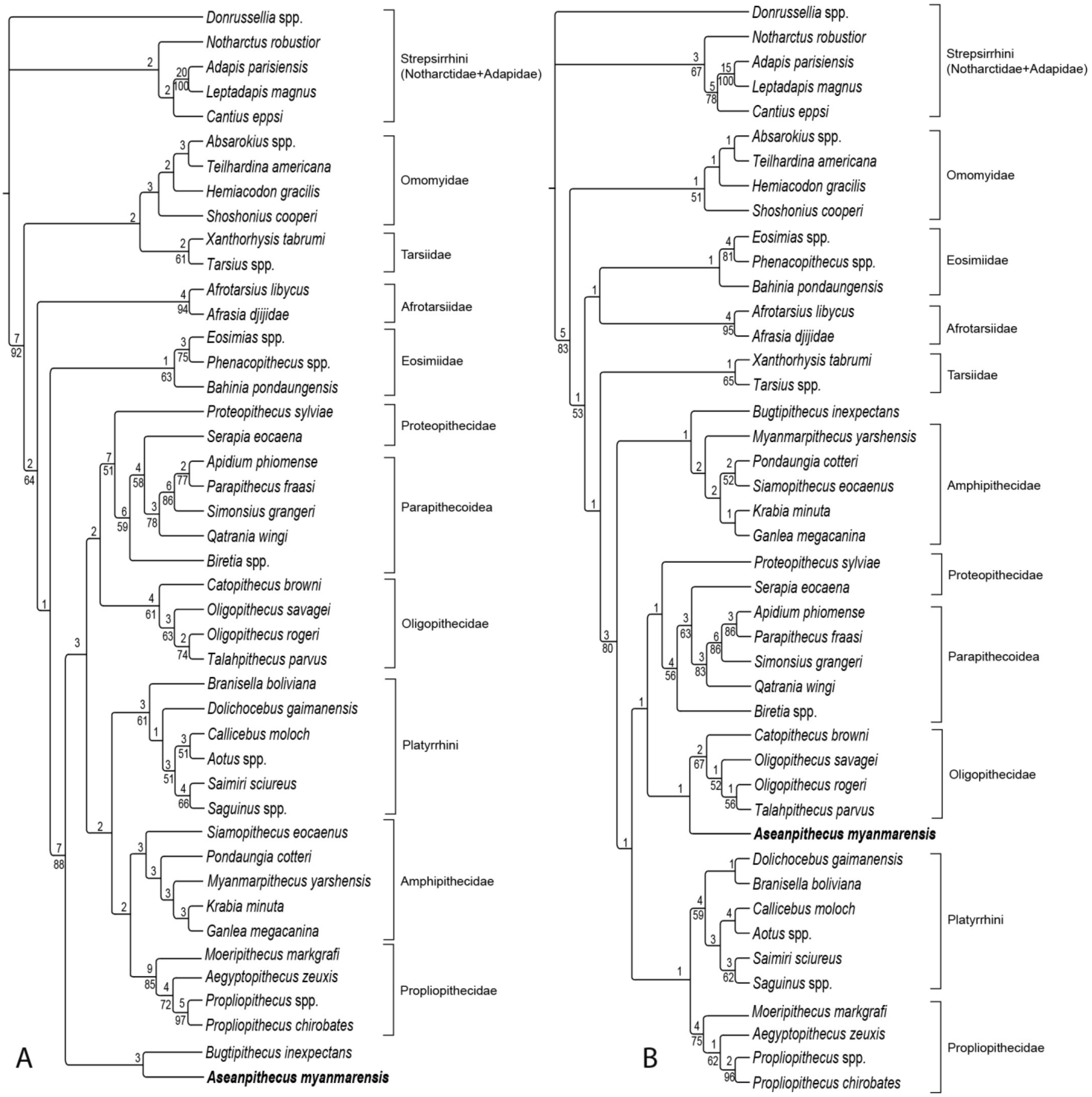

Supplementary Figure 5. A: single tree of 1477 steps obtained with analysis 3. B: single tree of 1375 steps obtained with analysis 4. Number above branches are Bremer support values. Number below branches are bootstrap support values obtained by a heuristic search with 1000 bootstrap replications (only values >50 are indicated).

Analysis 5 (topological constraint of ref.[9] enforced, *Phileosimias* retained, some multistate characters ordered) resulted in 8 most-parsimonious trees of 1502 steps (see Supplementary Fig. 6; Consistency index (CI) = 0.3209, Homoplasy index (HI) = 0.6791, CI excluding uninformative characters = 0.2999, HI excluding uninformative characters = 0.7001, Retention index (RI) = 0.5557, Rescaled consistency index (RC) = 0.1783) reconstructing *Aseanpithecus* as a basal member of the advanced anthropoids. *Aseanpithecus* is phylogenetically close to *Phileosimias* but no monophyletic group between these two taxa is supported by this analysis. The treatment of all multistate characters as unordered results in a poorly constrained tree in this configuration.

Analysis 6 (topological constraint of ref.[9] enforced, *Phileosimias* discarded, some multistate characters ordered) resulted in two equi-parsimonious trees of 1481 steps (see Supplementary Fig. 6; CI=0.3255, RI=0.5580, HI=0.6745, CI excluding uninformative characters= 0.3043, HI excluding uninformative characters = 0.6957, RC=0.1816) reconstructing *Aseanpithecus* as a basal member in the clade composed of the advanced anthropoids. The trees obtained in analyses 5 and 6 are very close to the topology obtained in analysis 3. The treatment of all multistate characters as unordered results in a most similar tree (only positions of eosimiids and afrotarsiids are inverted).

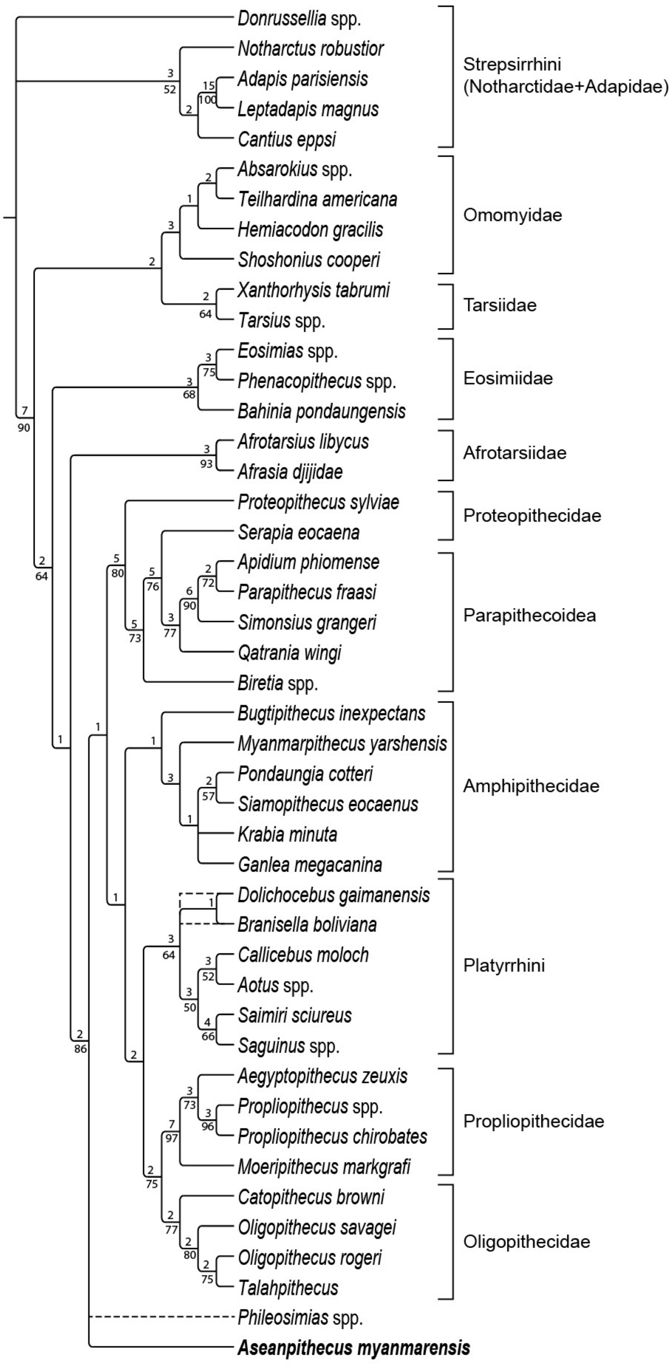

Supplementary Figure 6. Consensus tree of analyses 5 and 6 (analyses with topological constraint of ref.[9]). Dashed lines correspond to topology obtained in analysis 5. Number above branches are Bremer support values of analysis 6. Number below branches are bootstrap support values obtained by a heuristic search with 1000 bootstrap replications (only values >50 are indicated) for analysis 6.

Analysis 7 (topological constraint of ref.[10] enforced, *Phileosimias* conserved, all characters treated as unordered) resulted in two equiparsimonious trees of 1396 steps (see Supplementary Fig. 7A; CI = 0.3431, HI) = 0.6569, CI excluding uninformative characters = 0.3212, HI excluding uninformative characters = 0.6788, RI = 0.5413, RC = 0.1857) reconstructing again *Aseanpithecus* as a member of the clade of advanced anthropoids. *Aseanpithecus* has here, like for analyses 1 and 2, a less basal position than the Oligopithecidae which represent the most basal group within the clade of advanced anthropoids. *Aseanpithecus* belong to a clade composed of *Bugtipithecus* and the Amphipithecidae. This clade is the sister-group of crown anthropoids composed here of the Parapithecoidea, the Proteopithecidae, the Propliopithecidae and the Platyrrhini.

Note that, in this analysis, *Aseanpithecus*, *Bugtipithecus* and the Amphipithecidae are all more deeply nested within advanced anthropoids than the Oligopithecidae although our topological constraint did not specify the relative position between these taxa.

Analysis 8 (topological constraint of ref.[10] enforced, *Phileosimias* discarded, some multistate characters treated as ordered) resulted in two most-parsimonious trees of 1483 steps (Supplementary Fig. 7B; CI = 0.3250, HI = 0.6750, CI excluding uninformative characters = 0.3039, HI excluding uninformative characters = 0.6961, RI = 0.5571, RC = 0.1811). *Aseanpithecus* is forms a clade with *Bugtipithecus*, this clade being sister-group of crown anthropoids here composed of platyrrhines, amphipithecids, parapithecoids, proteopithecids and propliopithecids. Like for analysis 8, the oligopithecids have a more basal position than *Aseanpithecus* in the clade of advanced anthropoids.

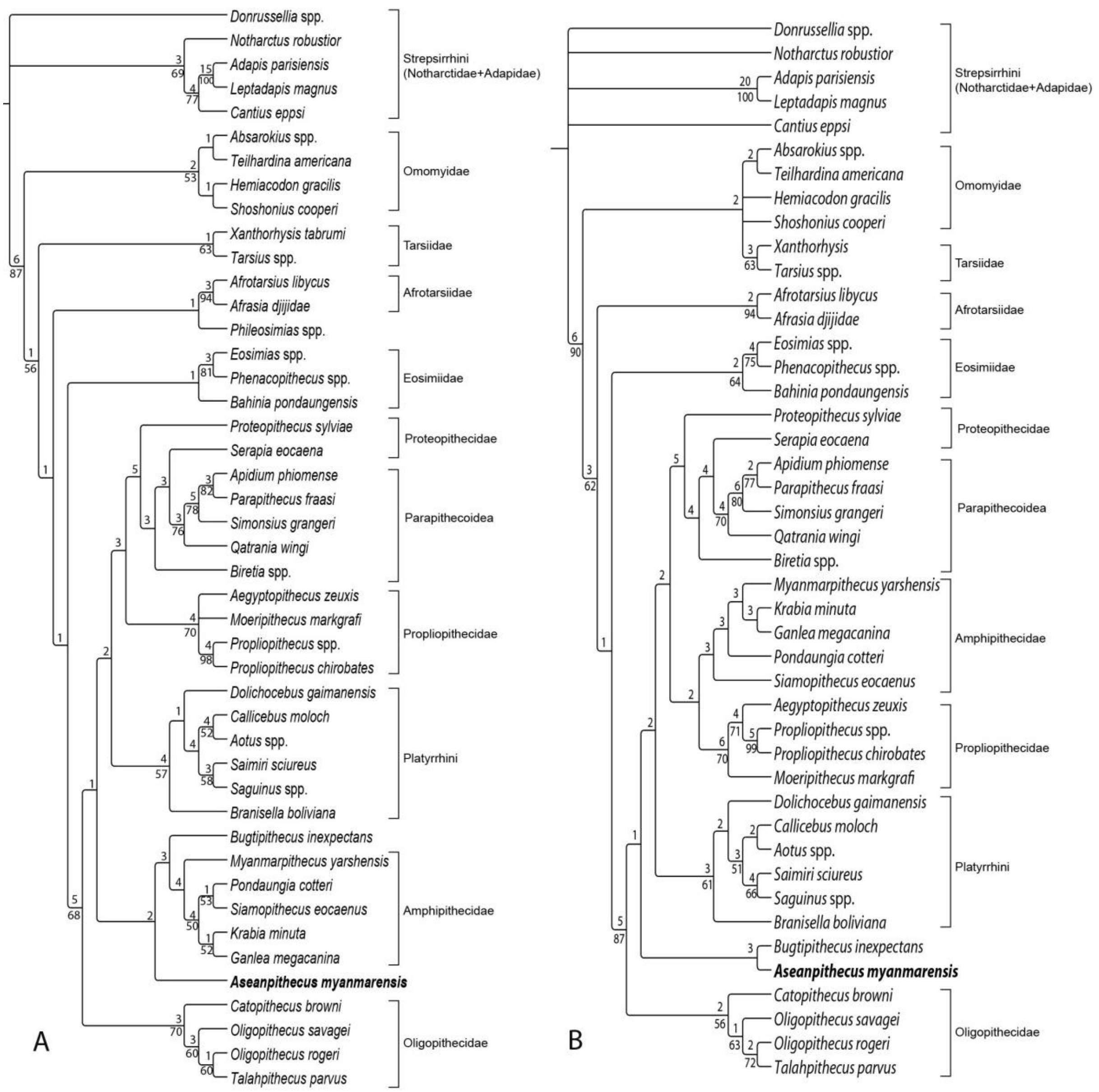

Supplementary Figure 7. A: strict consensus of two trees of 1396 steps obtained with analysis 7. B: strict consensus of two trees of 1483 steps obtained with analysis 8. Topological constraint of ref.[10] enforced. Number above branches are Bremer support values. Number below branches are bootstrap support values obtained by a heuristic search with 1000 bootstrap replications (only values > or =50 are indicated).

List of selected characters for the cladistic Analysis (324 characters)

Dental, cranial, and postcranial characters and character states used in the phylogenetic analysis. Characters followed by an asterisk are considered “ordered”. Some characters have been modified (different character state interpretation) from the original works (see Character references). These characters have been labeled by “ ‘ ” or “ “ ”. A score of “ ? ” is used if information is unavailable due to a lack of material. '-' is used if the character does not apply to a particular taxon. Tooth areas are calculated as the product of mesiodistal length and buccolingual breadth.

New characters, modified characters, coding changes compared with previous dataset

- Compared with previous versions of the data matrix, the characters OC1 to OC 6 have been newly coded.
- Character p9'[7] has been included in the matrix.
- Character p45 has been splitted into two new characters named p45a and p45b.
- Characters p33, P1, P7, P12 P18, ML151 and Cr32 have been modified and are now labeled by “ ’ ”.
- This version of the datamatrix contains coding changes compared with previous versions for characters i4, i17, p3, p9”, p13, p15, p20, p25, p40, p44, m8’, m9’, m10, m11, m8910, m13, m19, m20, m28, m29, m30, m41, m45, m55, I7, P4’, P6, P8, P9, P13’, M3, M10, M12’, M13, M15, M17”, M24, ML153, ML161, ML164, ML165, ML166, ML168; ML169, MLN, Cr16, Cr19, Cr26, Cr50, Cr51, H7.

Lower Teeth:

Incisors

1. i1*. Lower incisor number: 0 = three; 1 = two; 2 = one: I_1 present, I_2 absent; 3 = lower incisors absent.
2. i2. Lower incisor occlusal arrangement: 0 = arcuate battery from lateral perspective (U-shaped arcade); 1 = cusp tips staggered (V-shaped arcade).
3. i3. Lower incisor crown spacing: 0 = no spaces; 1 = spaces present between crowns.
4. i4. I_2-C diastema: 0 = present; 1 = absent.
5. i5*. I_{1-2} size (ratio of I_{1-2} area to M_1 area): 0 = very small (≤ 0.69); 1 = moderate sized ($\geq 0.70, \leq 1.07$); 2 = large (> 1.07).
6. i6*. I_1:I_2 proportions (ratio of I_1 area to I_2 area): 0 = I_1 much smaller than I_2 (< 0.65); 1 = I_1 smaller than I_2 ($\geq 0.65, < 0.82$); 2 = I_1 almost as large as I_2 ($\geq 0.83, < 1.00$); 3 = $I_1 > I_2$ ($\geq 1.01, < 1.25$); 4 = $I_1 >> I_2$ (≥ 1.25).

7. i7*. I_1 crown width (spatulate incisors only): 0 = considerably wider (m-d) than root (spatulate): 1 = narrow at apex, wider than root; 2 = “styliform” (crown apex approximately the same width as the cervical margin).
8. i8. I_2 crown cross-sectional shape (ratio of m-d length to b-l breadth): 0 = rounded oval (≥ 0.64); 1 = mesiodistally compressed (< 0.64).
9. i9*. Lower incisors crown height (crown heights judged from cementoenamel junction to crown tip on the buccal surface): 0 = low crowned; 1 = moderately high crowned; 2 = high crowned.
10. i11*. Lower incisor roots: 0 = erect or vertical; 1 = slightly procumbent; 2 = very procumbent.
11. i12*. Lower incisor crowns: 0 = erect or vertical; 1 = procumbent; 2 = very procumbent.
12. i14. I_1 crown shape: 0 = spatulate; 1 = lanceolate, pointed.
13. i15. I_2 heel development (a lingual swelling at the base of crown): 0 = heel absent; 1 = heel present.
14. i17*. Lower first incisor lingual cingulum: 0 = absent to weak; 1 = strong but incomplete; 2 = strong and complete.
15. i19*. Relative size of I_1 to M_1 (based on occlusal areas): 0 = I_1 very small ($I_1 << M_1$); 1 = moderately enlarged (I_1 < or = M_1); 2 = grossly enlarged ($I_1 > M_1$).

Canines

16. c1*. Female C_1 cross-sectional area relative to molar cross sectional area: 0 = very small ($C_1/M_1 < 0.40$); 1 = moderate (≥ 0.4, < 0.80); 2 = large (≥ 0.80, ≤ 1.20); very large (≥ 1.20)
17. c2*. $C_1/_1$ dimorphism (square root of male C_1 area/square root of female C_1 area): 0 = low (< 1.07); 1 = moderate (≥ 1.07, < 1.17); 2 = high (≥ 1.17).
18. c3. C_1 cross-sectional shape: 0 = rounded oval; 1 = mesiodistally compressed; 2 = buccolingually compressed.
19. c4. C_1 lingual crest development: 0 = rounded; 1 = sharp.
20. c5. Canine paracristid (not scored if species has canine incorporated into a tooth comb): 0 = oblique to occlusal plane; 1 = nearly horizontal to occlusal plane; 2 = forms part of cropping mechanism with $I_{1-2.}$
21. c6. Canine height (females): 0 = low, squat; 1 = narrow, short; 2 = tall, at or above tooth row.
22. ML18*. Lower canine crown: 0 = erect or vertical; 1 = procumbent; 2 = very procumbent.
23. ML19*. Lower canine root: 0 = erect or vertical; 1 = slightly procumbent; 2 = very procumbent.

Premolars

24. p1. $P^1/_1$: 0 = present; 1 = absent
25. p2. P_2: 0 = present; 1 = absent.
26. OC1.Relative size of P_2 and P_3: 0: $P_2 << P_3$ (ratio<0.6); 1: $P_2 < P_3$ (0.6<ratio<0.9); 2: P_2> or = P_3 (ratio >0.9)
27. OC2. Relative size of P_2 and P^2 : 0: P^2 larger or equal to P_2; 1: P^2 smaller than P_2
28. p3. P_2 roots: 0 = single; 1 = double.
29. p4’. P_3 roots: 0 = single; 1 = double.
30. p4”. P_4 roots: 0 = single; 1 = double.
31. p5*. Premolar crowding (overlapping of crowns): 0 = no crowding; 1 = slightly crowded; 2 = very crowded—mesial root positioned buccal to distal root.
32. p6*. P_3 paraconid: 0 = large; 1 = small; 2 = absent or extremely small.
33. p7*. P_4 paraconid: 0 = large; 1 = small; 2 = absent or extremely small.

34. p9'. P_4 paraconid position (labiolingually). 0 = 'mesial to protoconid'; 1 = 'mesiolingual, between protoconid and metaconid'; 2 = 'mesial to metaconid'.
35. p9''. P4 paraconid position (mesiodistally): 0 = widely spaced from the metaconid ; 1 = twinned with metaconid.
36. p11*. P_{3-4} cristid obliqua: 0 = absent; 1 = weak; 2 = strong.
37. p13. P_2 protoconid height and shape: 0 = slender, projects above protoconids of P_{3-4}; 1 = massive, projects above protoconids of P_{3-4}; 2 = not projecting, in line with P_3; 3 = extremely short, shorter than P_3.
38. p14. P_4 metaconid position: 0 = close to protoconid; 1 = widely spaced from protoconid.
39. p15. P_2 metaconid size: 0 = absent or trace; 1 = small.
40. p16*. P_3 metaconid size: 0 = absent or trace; 1 = small; 2 = large (as big as protoconid).
41. p17*. P_4 metaconid size: 0 = absent or trace; 1 = small; 2 = large (as big as protoconid).
42. p18. P_4 trigonid—configuration of lingual wall: 0 = closed; 1 = open.
43. p19. P_3 entoconid and lingual talonid crest: 0 = absent; 1 = lingual talonid crest present but an entoconid does not stand out above it; 2 = entoconid forms a small discrete cusp.
44. p20. P_4 entoconid and lingual talonid crest: 0 = absent; 1 = lingual talonid crest present but an entoconid does not stand out above it; 2 = entoconid forms a small discrete cusp.
45. p21. P_4 lateral and medial protocristid: 0 = continuous between metaconid and protoconid; 1 = discontinuous between metaconid and protoconid.
46. p22. P_3 lateral protocristid orientation: 0 = transversely oriented; 1 = distolingually oriented; 2 = absent.
47. p23. P_4 lateral protocristid orientation: 0 = transversely oriented; 1 = distolingually oriented.
48. p24. P_{3-4} posterior trigonid wall: 0 = complete [taxa without metaconids are assigned this character state]; 1 = deeply notched.
49. p25. P_{3-4} hypoconid size: 0 = large; 1 = small or absent.
50. p26. P_{3-4} hypoconid (or distal terminus of oblique cristid) position: 0 = distal to protoconid; 1 = distal to metaconid, or between protoconid and metaconid
51. p27*. P_4 hypocristid shearing development: 0 = absent; 1 = weak; 2 = strong.
52. p28*. P_2 buccal cingulum development: 0 = absent; 1 = incomplete, broken at protoconid and hypoconid; 2 = complete.
53. p29*. Lower premolar inflation: 0 = not basally inflated; 1 = slightly basally inflated; 2 = very basally inflated.
54. p30*. P_4 exodaenodonty: 0 = not exodaenodont; 1 = slightly exodaenodont; 2 = very exodaenodont.
55. p31*. P_4 talonid length (ratio of midline m-d length of trigonid to m-d length of talonid): 0 = extremely short or non-existent (tri:tal ≥ 1.61); 1 = short (much shorter than trigonid) (tri:tal ≥ 1.27, < 1.61); 2 = equal or slightly shorter in length to trigonid (tri:tal ≥ 0.92, < 1.27); 3 = talonid longer than trigonid (tri:tal < 0.91).
56. p33'*. Premolar orientation: 0 = Crown bases vertical in lateral perspective; 1 = slightly oblique; 2 = strongly oblique, due to a strong mesial projection of the crown.
57. p34. P_4 anterobuccal cingulum development: 0 = absent or trace; 1 = strong.
58. p36*. P_4 postprotoconid ridge: 0 = weak or absent; 1 = moderate; 2 = very strong.
59. p37*. P_4 postmetaconid ridge: 0 = weak or absent; 1 = moderate; 2 = very strong.
60. p40*. P_4 paraconid height: 0 = low; 1 = moderate; 2 = high (nearly as high as protoconid).
61. p41*. P_{3-4} protoconid height: 0 = P_3 much lower than P_4; 1 = P_3 slightly lower than P_4; 2 = P_3 equal in height to P_4; 3 = P_3 higher than P_4.
62. p42*. P_3 to P_4 area: 0 = 0.45-0.59; 1 = 0.60-0.69; 2 = 0.70-0.79; 3 ≥ 0.80.

63. p43*. P_4 m-d L/ b-l W: 0 = (< 0.95); 1 = (≥ 0.96, < 1.14); 2 = (≥ 1.15, < 1.20); 3 = (≥ 1.21, < 1.35; 4 = (≥ 1.36, < 1.46); 5 = (> 1.47).
64. p44*. Ratio of P_4 area to M_1 area: 0 = (< 0.62); 1 = (≥ 0.63, < 0.72); 2 = (≥ 0.73, < 0.82); 3 = (≥ 0.83, < 0.92); 4 = (≥ 0.93, < 1.02); 5 = (> 1.03).
65. p45a. P/3 root orientation: 0 = aligned mesiodistally; 1 = shifted laterally
66. p45b. P/4 root orientation: 0 = aligned mesiodistally; 1 = mesial root shifted buccally

Molars
67. m4. M_3 root number: 0 = one; 1 = two.
68. m6*. M_2 trigonid width (ratio of buccolingual breadths of trigonid and talonid): 0 = much wider than talonid (≥ 1.11); 1 = widths similar (< 1.11, > 0.90); 2 = much narrower than talonid (≤ 0.90).
69. m7*. M_3 trigonid width (based on relative buccolingual breadths): 0 = much wider than talonid (≥ 1.20); 1 = trigonid and talonid widths similar (≤ 1.20-1.05); 2 = trigonid narrower than talonid (< 1.05).
70. OC3. M_1 paraconid: 0 = present; 1 = absent
71. m8'. M_1 paraconid position: 0 = mesiolingual, between protoconid and metaconid; 1 = mesial to metaconid.
72. m9'. M_2 paraconid position: 0 = mesiolingual, between protoconid and metaconid; 1 = mesial to metaconid.
73. OC4. M_3 paraconid : 0 = present; 1 = absent
74. m10. M_3 paraconid position: 0 = mesiolingual, between protoconid and metaconid; 1 = mesial to metaconid.
75. m8-9-10'. $M_{2\text{-}3}$ paraconid location: 0 = widely spaced from the metaconid; 1 = twinned with metaconid.
76. m11. M_1 parastylid (= premetacristid): 0 = absent; 1 = present.
77. m12*. Molar metastylids (postmetacristids): 0 = absent; 1 = small; 2 = large.
78. m13. M_3 hypoconulid: 0 = single; 1 = double
79. m14*. M_3 heel: 0 = absent; 1 = narrower than talonid; 2 = approximately equal in width to talonid.
80. m15*. Molar enamel surface: 0 = smooth; 1 = slightly crenulated; 2 = highly crenulated.
81. m16*. M_1 trigonid height (ratio of trigonid height to talonid height measured on the buccal aspect of the crown): 0 = higher than talonid (≥ 1.20); 1 = slightly higher than talonid (≥ 1.10, < 1.20); 2 = trigonid and talonid of similar height (< 1.10).
82. m17. $M_{1\text{-}2}$ cusp relief: 0 = moderate to high; 1 = low.
83. m18. M_1 trigonid lingual configuration: 0 = open; 1 = closed.
84. m19. M_1 metaconid position: 0 = transversely aligned—lingual to protoconid; 1 = slightly distolingual to protoconid.
85. m20*. M_2 paraconid development: 0 = absent; 1 = small; 2 = large.
86. m21. $M_{1\text{-}2}$ lateral protocristid orientation: 0 = runs toward metaconid; 1 = runs toward hypoflexid.
87. m22. M_1 distal trigonid wall: 0 = complete; 1 = deeply notched by protoconid/metaconid sulcus; 2 = medial and lateral protocristid do not meet but no sulcus is visible.
88. m23. M_2 distal trigonid wall: 0 = complete; 1 = deeply notched by protoconid/ metaconid sulcus; 2 = medial and lateral protocristid do not meet but no sulcus is visible.
89. m24. $M_{1\text{-}3}$ wear facet X: 0 = present; 1 = absent.
90. m25*. $M_{1\text{-}2}$ entoconid: 0 = barely stands out on lingual talonid marginal crest; 1 = a small discrete cusp; 2 = a large cusp.
91. m26*. $M_{1\text{-}2}$ postentoconid sulcus: 0 = prominent; 1 = faintly visible; 2 = absent.
92. m27*. M_1 hypoconulid size: 0 = large; 1 = moderate; 2 = small; 3 = absent.

93. m28*. M_2 hypoconulid size: 0 = large; 1 = moderate; 2 = small; 3 = absent.
94. m29*. M_3 hypoconulid size: 0 = large; 1 = moderate; 2 = small; 3 = absent.
95. m30*. M_{1-2} hypoconulid position: 0 = twinned to entoconid; 1 = near midline; 2 = slightly buccal to midline.
96. m31*. M_{1-2} cristid obliqua development: 0 = weak (rounded); 1 = strong (trenchant); 2 = very strong (trenchant).
97. m32*. M_1 cristid obliqua orientation: 0 = reaches trigonid wall at a point distal to protoconid; 1 = reaches trigonid wall at a point distolingual to protoconid; 2 = reaches trigonid wall at a point distal to metaconid.
98. m33*. M_2 cristid obliqua orientation: 0 = reaches trigonid wall at a point distal to protoconid; 1 = reaches trigonid wall at a point distolingual to protoconid; 2 = reaches trigonid wall at a point distal to metaconid.
99. m34. M_1 cristid obliqua terminus: 0 = runs to base of trigonid; 1 = runs part way up the distal trigonid wall; 2 = connects with protoconid tip or protocristid; 3 = connects with metaconid.
100. m35. M_2 cristid obliqua terminus: 0 = runs to base of trigonid; 1 = runs part way up the distal trigonid wall; 2 = connects with protoconid tip or protocristid; 3 = connects with metaconid.
101. m36. M_3 cristid obliqua terminus: 0 = runs to base of trigonid; 1 = runs part way up the distal trigonid wall; 2 = connects with protoconid tip or protocristid; 3 = connects with metaconid.
102. m37. M_{1-2} centroconid development: 0 = present; 1 = absent.
103. m38*. M_{1-2} hypocristid development: 0 = absent or seen only as a trace; 1 = weak; 2 = strong.
104. m39*. M_3 hypocristid development: 0 = absent or seen only as a trace; 1 = weak; 2 = strong.
105. m40*. Lingual configuration of M_{1-2} talonid: 0 = open; 1 = notched lingually but not open; 2 = closed.
106. m41. M_{1-2} distal fovea: 0 = absent; 1 = present (weak); 2 = present (large).
107. m42. M_{1-2} hypocristid configuration: 0 = simple; 1 = with accessory cusp close to hypoconid.
108. m43. M_{1-2} cristid obliqua: 0 = notched; 1 = straight.
109. m44*. Molar cusp inflation: 0 = cusps not inflated, marginally positioned; 1 = slightly inflated; 2 = very inflated.
110. m45*. M_{1-2} buccal cingulum development: 0 = absent to trace; 1 = partial, broken at protoconid and hypoconid; 2 = complete.
111. m46*. M_1 hypoflexid depth: 0 = very shallow; 1 = moderate; 2 = deep.
112. m47*. M_2 hypoflexid depth: 0 = very shallow; 1 = moderate; 2 = deep.
113. m53*. Ratio of M_2 length to M_3 length: 0 = M_3 much longer than M_2 (0.71-0.80); 1 = M_3 longer than M_2 (0.81-0.90); 2 = M_3 equal than M_2 (0.91-1.00); 3 = M_3 smaller than M_2 (1.01-1.12); 4 = M_3 much smaller than M_2 ($\geq$ 1.13); 5 = if M_3 absent.
114. m55*. M_1 mesiodistal length/buccolingual breadth: 0 = 1.0-1.15; 1 = 1.16-1.22; 2 = 1.23-1.32; 3 = > 1.33.
115. m56. Convergence of buccal and lingual molar cusp walls: 0 = convergent; 1 = vertically sided.
116. m57. M_{1-2} entoconid position relative to hypoconid: 0 = transverse to hypoconid; 1 = distal to hypoconid.
117. ML88*. M_{1-3} Pre-entocristid: 0 = indistinct to absent; 1 = weakly developed (low); 2 = well-developed (strong and high).

Upper Teeth:

Incisors

118. I1*. I^1-I^2 interstitial contact: 0 = absent; teeth widely spaced; 1 = present as narrow contact; 2 = I^2 tightly packed against I^1, I^1 preparacrista abbreviated.

119. I2. I^1-I^1 interstitial contact: 0 = present; 1 = absent: a wide space occurs in the midline between these teeth.

120. I3. I^2-C diastema: 0 = present; 1 = absent.

121. I4*. I^1 area: I^2 area: 0 = areas approximately equal (≤ 1.00); 1 = I^1 slightly larger than I^2 (> 1.00, < 1.40); 2 = I^1 much larger than I^2 (> 1.40).

122. I5*. I^1 size (I^1 area: M^1 area): 0 = incisor small (≤ 0.50); 1 = incisor moderate (> 0.50, < 0.56); 2 = incisor large (≥ 0.56).

123. I6*. I^1 occlusal shape (mesiodistal length/buccolingual breadth): 0 = rounded oval (< 1.05); 1 = buccolingually compressed (> 1.05, < 1.30); 2 = extremely compressed (> 1.30).

124. I7*. I^2 occlusal shape (mesiodistal length /buccolingual breadth): 0 = rounded oval (≤ 1.05); 1 = slightly buccolingually compressed (> 1.05, < 1.30); 2 = extremely buccolingually compressed ≥ 1.30).

125. I10. I^1 occlusal edge orientation (for spatulate incisors only; all others scored as “?”): 0 = occlusal edge orthogonal to long axis of root; 1 = occlusal edge wears at a steep angle to long axis of root; 2 = crown with pronounced mesial asymmetry (= mesial process) in unworn state.

126. I11. I^{1-2} lingual cingulum: 0 = moderate, continuous; 1 = strong.

127. I12. I^1 basal lingual cusp: 0 = absent; 1 = present.

128. I13. I^1-I^2 buccal cingulum: 0 = absent; 1 = present.

Canines

129. C1. C^1 cross-sectional shape: 0 = oval; 1 = rounded.

130. C2*. Upper canine occlusion: 0 = C^1 wears against P_{1-2}; 1 = C^1 wears against P_2; 2 = C^1 wears against P_{2-3}; 3 = C^1 wears against P_3.

131. C3. C^1 mesial groove (females): 0 = shallow or absent; 1 = deep.

132. C4*. C^1 lingual cingulum: 0 = weak or absent; 1 = strong; 2 = very strong.

Premolars

133. OC5. P^2: 0 = present; 1 = absent.

134. P1’*. P^2 root number: 0 = one; 1 = two; 2 = three.

135. P2. P^3 root number: 0 = two; 1 = three.

136. P3. P^4 root number: 0 = two; 1 = three.

137. P4’*. Ratio of P^2 area to P^3 area: 0 = P^2 much smaller (≤ 0.85)); 1 = P^2 smaller (> 0.85, < 0.95); 2 = P^2 equal (≥ 0.95); 3 = clearly larger.

138. P5*. Ratio of P^4 area to M^1 area: 0 = P^4 << M^1 (≤ 0.66); 1 = P^4< M^1 (> 0.66, ≤ 0.76); 2 = P^4 = M^1 (0.77-1.05); 3 = P^4 > M^1 (> 1.06).

139. P6. P^2 occlusal outline: 0 = triangular; 1 = suboval with the long axis b-l; 2 = suboval with the long axis m-d; 3 = round.

140. OC6. P3/ Occlusal outline 0: = Suboval (with roughly mesiodistal long axis); 1 = Triangular (buccal lobe much wider than lingual lobe); 2 = Trapezoid (tooth buccolingually extended with buccal lobe wider than lingual lobe); 3 = Rectangular (tooth buccolingually extended with lingual lobe as wide as buccal lobe); 4 = Squared (tooth as wide as long with buccal lobe as wide as lingual lobe

141. P7’. P4 occlusal outline: 0 = triangular; 1 = trapezoid; 2 = rectangular; 3 = squared.

142. P8. P^{3-4} trigon/talon proportions: 0 = trigon > = talon; 1 = trigon < talon.

143. P9. P^3 protocone: 0 = present; 1 = absent.

144. P10. P^4 metacone: 0 = absent; 1 = present.
145. P11. P^4 protocone: 0 = low relative to paracone; 1 = high relative to paracone.
146. P12’. P2 protocone: 0 = present; 1 = absent
147. P13’. P^2 hypocone: 0 = absent; 1 = present·
148. P14*. P^4 paraconule: 0 = large; 1 = small; 2 = absent.
149. P15. $P^{3\text{-}4}$ parastyles: 0 = present; 1 = absent.
150. P16. $P^{3\text{-}4}$ metastyles: 0 = absent; 1 = present.
151. P17. $P^{3\text{-}4}$ postprotocrista: 0 =strong; 1 = weak, short.
152. P18’. $P^{2\text{-}4}$ distal crown margin: 0 = smoothly rounded; 1 = waisted between buccal and lingual cusps.
153. P19. $P^{3\text{-}4}$ lingual cingulum: 0 = absent or weak; 1 = strong.
154. P20. P^3 metacone: 0 = absent; 1 = present
155. P21. $P^{3\text{-}4}$ buccal cingulum development: 0 = absent or weak; 1 = strong.
156. ML126*. P^4 hypocone: 0 = minute to absent; 1 = present but small; 2 = strong.
157. ML127*. P^3 hypocone: 0 = minute to absent; 1 = present but small; 2 = strong.

Molars
158. M1*. $M^{1\text{-}2}$ root number: 0 = three, three; 1 = three, two; 2 = two, two.
159. M2*. M^3 root number: 0 = three; 1 = two; 2 = one.
160. M3*. M^2 shape (bl/md): 0 = very transverse (> 1.65); 1 = transverse (< 1.65, > 1.30); 2 = squared (≤ 1.30).
161. M4*. Ratio of M^1 area to M^2 area: 0 = $M^1 >> M^2$ (≥ 1.40); 1 = $M^1 > M^2$ (< 1.40, > 1.0); 2 = $M^1 \leq M^2$ (≤ 1.0).
162. M7’*. $M^{1\text{-}2}$ metaconule: 0 = absent; 1 = single; 2 = double.
163. M9*. $M^{1\text{-}2}$ preprotoconule: 0 = absent; 1 = weak; 2 = strong.
164. M10*. M^1 hypocone size: 0 = large; 1 = small; 2 = minute to absent.
165. M11*. M^2 hypocone size: 0 = large; 1 = small; 2 = minute to absent.
166. M12’*. $M^{1\text{-}2}$ hypocone position: 0 = distal, far lingual to protocone; 1 = distal, slightly lingual to protocone; 2 = distal, slightly buccal to protocone.
167. M13*. $M^{1\text{-}2}$ prehypocrista development: 0 = absent; 1 = weak; 2 = strong, reaches to postprotocrista, encloses the talon lingually.
168. M14. M^3 prehypocrista development: 0 = absent; 1 = strong, reaches to postprotocrista, encloses the talon lingually.
169. M15. M^1 or M^2 paraconule position: 0 = attached to preprotocrista; 1 = unattached to preprotocrista.
170. M16*. $M^{1\text{-}2}$ metaconule: 0 = absent to indistinct; 1 = small; 2 = moderate; 3 = large.
171. M17’*. $M^{1\text{-}2}$ mesostyle size: 0 = absent to indistinct; 1 = moderate; 2 = strong.
172. M17”. $M^{1\text{-}2}$ mesostyle position: 0 = attached to ectocrista; 1 = present on buccal cingulum.
173. M20*. P^4-M^1 pericone: 0 = absent; 1 = small; 2 = large.
174. M22*. $M^{1\text{-}3}$ lingual cingulum development: 0 = absent to indistinct; 1 = weak, broken; 2 = strong, complete.
175. M24*. $M^{1\text{-}2}$ buccal cingulum development: 0 = absent to indistinct; 1 = weak; 2 = strong.
176. M27. $M^{1\text{-}2}$ pre-metaconule cristae: 0 = absent or weak; 1 = strong
177. M28. $M^{1\text{-}2}$ post-metaconule cristae: 0 = absent or weak; 1 = strong
178. M30*. M^3 paraconule: 0 = absent; 1 = small-moderate; 2 = large
179. M31*. Molar protocone lingual inflation: 0 = not inflated; 1 = slightly inflated; 2 = very inflated.
180. M33*. M^2 buccal expansion of paracone (specify which tooth): 0 = no expansion; 1 = slight expansion; 2 = considerable expansion.

181. M34*. M^3 metacone: 0 = absent or very small; 1 = moderate (but smaller than paracone; 2 = large (equal to paracone).
182. M36*. M^3 hypocone: 0 = absent or very small; 1 = small; 2 = large.
183. M37*. M^1 paraconule size: 0 = absent; 1 = small-moderate (smaller than paracone); 2 = large (nearly as large as or larger than paracone).
184. M44*. M^{1-3} anterior cingulum: 0 = strong, complete, long (connected to parastyle); 1 = strong, short; 2 = weak or absent.
185. M46*. M^3 size relative to M^1: 0 = very small (half the size of M^1 or less); 1 = small (two thirds); 2 = large (approximately as large).
186. ML147*. M^{1-2} metastyle: 0 = indistinct to absent; 1 = moderate; 2 = strong.
187. ML148*. M^{1-2} parastyle: 0 = indistinct to absent; 1 = moderate; 2 = strong.
188. ML149. M^{1-2} parastyle position: 0 = mesial to paracone; 1 = mesiobuccal to paracone.
189. ML150. M^{1-2} metastyle position: 0 = distal to metacone; 1 = distobuccal to metacone.
190. ML151. M^{1-3} posterior cingulum: 0 = moderate, does not reach the metastyle / the distobuccal corner of the tooth; 1 = connected to metastyle / reaches the distobuccal corner of the tooth.
191. ML152*. M^{1-3} posterior margin (waisted between buccal and lingual cusps): 0 = indistinct to absent; 1 = present but shallow; 2 = present, deep.
192. ML153*. M^{1-2} postparacrista: 0 = indistinct to absent; 1 = weakly developed; 2 = well developed (but well-marked notch between postparacrista and premetacrista); 3 = strongly elevated (weak notch between postparacrista and premetacrista).
193. ML154*. M^{1-2} premetacrista: 0 = indistinct to absent; 1 = weakly developed; 2 = well developed (but well-marked notch between premetacrista and postparacrista); 3 = strongly elevated (weak notch between premetacrista and postparacrista).
194. ML155. M^{1-3} protocone arrangement: 0 = normal position; 1 = oblique.
195. ML156. M^{1-2} postprotocrista development: 0 = strong; 1 = tiny.
196. ML157*. M^1 postprotocrista length: 0 = indistinct to absent; 1 = short; 2 = long.
197. ML158*. M^2 postprotocrista length: 0 = indistinct to absent; 1 = short; 2 = long.
198. ML159. M^1 postprotocrista direction: 0 = transverse, directed toward metaconule (or virtual metaconule emplacement); 1 = lateral, directed toward the lingual posterior cingulum (post-protocone fold-like).
199. ML160. M^2 postprotocrista direction: 0 = transverse, directed toward metaconule (or virtual metaconule emplacement); 1 = lateral, directed toward lingual posterior cingulum (post-protocone fold-like).
200. ML161. M^1 postprotocrista terminus: 0 = runs to base of metacone (with hypometacrista); 1 = runs to metaconule (at the level of the small or virtual metaconule); 2 = runs to posterior cingulum; 3 = limited at a point distal to protocone.
201. ML162. M^2 postprotocrista terminus: 0 = runs to base of metacone (with hypometacrista); 1 = runs to metaconule (at the level of the small or virtual metaconule); 2 = runs to posterior cingulum; 3 = limited at a point distal to protocone.
202. ML163. M^{1-2} preprotocrista: 0 = low; 1 = elevated.
203. ML164. M^1 preprotocrista connection (buccal side): 0 = connected to parastyle (by way of preparaconule crista); 1 = connected to paraconule (or near to it or to a virtual paraconule).
204. ML165. M^2 preprotocrista connection (buccal side): 0 = connected to parastyle (by way of preparaconule crista); 1 = connected to paraconule (or near to it or to a virtual paraconule).
205. ML166*. M^{1-2} postparaconule crista: 0 = indistinct to absent; 1 = moderate; 2 = well-developed (connected to paracone).

206. ML168*. M^{1-2} hypometacrista: 0 = absent; 1 = weakly developed (low and short); 2 = well-developed (high).
207. ML169*. M^{1-2} hypoparacrista: 0 = absent; 1 = weakly developed (short); 2 = well-developed (high).
208. MLN*. Hypometaconulecrista: 0 = indistinct to absent; 1 = moderate (not connected to protocone); 2 = well-developed (connected to protocone or postprotocrista).

Cranial characters:
209. Cr 1. Transverse septum arising from the cochlear housing: 0 = absent; 1 = present and forming the lateral wall of an anterior accessory cavity pneumatized from the tympanic cavity; 2 = present and forming the lateral wall of an anterior accessory cavity pneumatized from the epitympanic recess.
210. Cr 2. Extent of pneumatization of anterior accessory cavity: 0 = Anterior accessory cavity lies anterior to the tympanic cavity and is not trabeculated; 1 = anterior accessory cavity extends medial to the tympanic cavity, and is trabeculated.
211. Cr 3. Pneumatization of mastoid (from epitympanic recess?): 0 = absent; 1 = present.
212. Cr 4. Presence or absence of perbullar pathway: 0 = absent; 1 = present and formed exclusively by the petrosal bone.
213. Cr 5. Anteroposterior location of posterior carotid foramen in bulla: 0 = Posterior to line joining midpoints of tympanic bones; 1 = anterior to this line.
214. Cr 6*. Mediolateral position of posterior carotid foramen in bulla: 0 = medial; 1 = midline of the bulla; 2 = lateral.
215. Cr 7. Ventrodorsal position of the carotid foramen in the bulla: 0 = dorsal, adjacent to basioccipital or mastoid bone; 1 = ventral.
216. Cr 8*. Position of posterior carotid foramen relative to fenestra cochleae: 0 = posterior; 1 = ventral; 2 = anterior.
217. Cr 9. Position of the internal carotid canal relative to the fenestra cochleae: 0 = runs across ventral lip of the fenestra cochleae, shielding it from ventral view when a canal is present; 1 = internal carotid canal does not shield the fenestra cochleae from ventral view.
218. Cr 10. Position of the portion of the internal carotid/promontory artery (or its accompanying nerves) lying on the promontorium anterior to the fenestra cochleae: 0 = on ventrolateral surface of promontorium; 1 = contacting only the cupula of the cochlea.
219. Cr 11. Size of stapedial and promontory canals: 0 = both stapedial and promontory canals are large; 1 = stapedial slightly smaller than promontory; 2 = stapedial highly reduced or absent altogether; 3 = stapedial larger than promontory; 4 = both promontory and stapedial canals absent.
220. Cr 12. Morphology of promontory canal, when present: 0 = open trough; 1 = complete canal.
221. Cr 14. Position of ventral edge of the tympanic bone: 0 = intrabullar, or aphaneric; 1 = extrabullar or phaneric.
222. Cr 15. The shape of the tympanic bone: 0 = ribbon-like or only slightly expanded; 1 = laterally expanded into a collar or tube: ? = due to fusion with surrounding bones, of unknown shape.
223. Cr 16. Morphology of annular bridge: ? = This character is not analyzable in those taxa with an extrabullar tympanic, or those in which this region is not known; 0 = Linea semicircularis or partial anular bridge formed on a entotympanic bulla; 1 = linea semicircularis formed on a petrosal bulla; 2 = a complete annular bridge.
224. Cr 17. Encroachment of the auditory bulla on the pterygoid fossa: 0 = absent; 1 = present and formed by anterior accessory cavity; 2 = present and formed by the tympanic cavity.

225. Cr 18. Nature of contact between the lateral pterygoid plate and the bulla wall: 0 = absent; 1 = laminar; 2 = abutting.
226. Cr 19. Extent of contact between the lateral pterygoid plate and the bulla wall: 0 = slight; 1 = or very extensive.
227. Cr 20. Flange of basioccipital overlapping medial bulla wall: 0 = absent or minimal; 1 = extensive.
228. Cr 21. Suprameatal foramen: 0 = absent; 1 = present, small and in the posterior root of the zygomatic arch; 2 = present, large, and above the external auditory meatus.
229. Cr 22. Patent parotic fissure: 0 = present; 1 = absent.
230. Cr 23*. Size of orbits: 0 = small; 1 = large; 2 = extremely large.
231. Cr 24*. Postorbital closure: 0 = none; 1 = postorbital bar present; 2 = postorbital septum present.
232. Cr 25. Composition of the postorbital septum: 0 = zygomatic forms most of the septum; 1 = frontal forms most of the septum.
233. Cr 26. Zygomatic-lacrimal contact: 0 = present; 1 = absent.
234. Cr 27. Pronounced interorbital constriction: 0 = absent; 1 = present below olfactory tract.
235. Cr 28. Contact between lacrimal and palatine: 0 = present; 1 = separated by a large fronto-maxillary contact (and in some taxa, a small os planum of the ethmoid); 2 = separated by a large os planum.
236. Cr 29. Foramen rotundum: 0 = absent; 1 = present.
237. Cr 30. Position of lacrimal foramen: 0 = outside orbital margin; 1 = within the orbit or on the rim.
238. Cr 31. Metopic suture in adult: 0 = unfused; 1 = fused.
239. Cr 32'. Orbital convergence: 0 = low (less convergent than primates); 1 = moderate; 2 = high; 3 = very high.
240. Cr 33*. Posterior nasal spine: 0 = reduced or absent; 1 = small but distinct; 2 = robust and long
241. Cr 34. Posterior palatine torus: 0 = present; 1 = absent.
242. Cr 35. Pyramidal processes: 0 = medially placed; 1 = laterally placed.
243. Cr 36*. Length of medial pterygoid plate: 0 = long medial pterygoid plate extending one-third to one half of the distance to the anterior surface of the bulla; 1 = short but distinct from lateral pterygoid plate for its entire dorsoventral extent; 2 = medial pterygoid plate entirely absent, or reduced to a low rugosity.
244. Cr 37. Snout length: 0 = long snouts; 1 = short snouts.
245. Cr 38. Maxillary depth: 0 = deep; 1 = shallow.
246. Cr 39. Complete symphyseal fusion: 0 = absent; 1 = present.
247. Cr 40. Temporomandibular joint morphology: 0 = biconcave and transversely wide; 1 = anteroposteriorly oriented trough.
248. Cr 41. Entoglenoid process morphology: 0 = weak or absent; 1 = strong.
249. Cr 42. Inter-incisor diastema width: 0 = broad and wider than that of extant haplorhines; 1 = narrow, haplorhine-like.
250. Cr43. Coronoid height relative to condyle: 0 = very far above; 1 = slightly above or equal.
251. Cr44*. Condyle height relative to toothrow: 0 = at level of tooth row; 1 = slightly above; 2 = well above tooth row.
252. Cr45. Corpus robusticity: 0 = shallow; 1 = deep.
253. Cr46. Zygomatico-parietal contact at pterion: 0 = no postorbital closure; 1 = zygomatico-parietal contact; 2 = alisphenoid-frontal contact.
254. Cr48. Epitympanic crest: 0 = absent; 1 = present.

255. Cr49. Broad ascending wing of premaxilla: 0 = narrow; 1 = broad.
256. Cr 50/301. Basioccipital stem: 0 = narrow; 1 = broad.
257. Cr51/302. Choanal shape: 0 = narrow; 1 = broad.
258. Cr52/292. Orientation of the mandibular symphysis: 0 = symphysis procumbent; 1 = symphysis erect.

Postcranial characters:

Humerus

259. H1*. Shape of distal edge of the humeral trochlea: 0 = cylinder, distal edge perpendicular to shaft; 1 = distal edge somewhat angled to shaft; 2 = distal edge very angled.
260. H2. Relative heights of medial and lateral edges of humeral trochlea: 0 = subequal; 1 = medial edge more flared than lateral edge.
261. H3*. Trochleocapitular ridge: 0 = absent; 1 = weak but distinct; 2 = moderately distinct; 3 = very distinct.
262. H4. Waisted trochlea (Minimum trochlear diameter/maximum trochlear diameter x 100): 0 = > 70 (unwaisted); 1 = ≤ 70 (waisted).
263. H5*. Width of capitulum relative to trochlea (100 x ventral capitulum width/ventral trochlear width): 0 = < 100; 1 = between 100 and 140; 2 = 140-200; 3 = greater than 200.
264. H6. Entepicondylar foramen: 0 = present; 1 = variable; 2 = absent .
265. H7. Entepicondylar foramen position: 0 = above medial epicondyle; 1 = above ventral trochlea; 2 = above dorsal trochlea.
266. H8. Medial epicondyle size: 0 = reduced; 1 = prominent.
267. H9. Dorsal placement of medial epicondyle: 0 = parallel; 1 = slight dorsal; 2 = large dorsal angle.
268. H10'*. Shape of the lateral edge of the dorsal trochlea: 0 = not pronounced; 1 = moderately pronounced; 2 = very pronounced.
269. H10"*. Shape of the medial edge of the dorsal trochlea: 0 = not pronounced; 1 = moderately pronounced; 2 = very pronounced.
270. H11*. Dorsoepitrochlear fossa: 0 = present (strong); 1 = small, shallow; 2 = absent.
271. H12*. Olecranon fossa shape: 0 = shallow; 1 = moderate; 2 = deep.
272. H14*. Brachialis flange; 0 = broad; 1 = moderate; 2 = narrow.
273. H15. Bicipital groove morphology: 0 = shallow; 1 = deep.
274. H18/. Capitular tail: 0 = ventral articular wdth <2.5 times the ventral capitular width; 1 = ventral articular wdth > 2.5 times the ventral capitular width.
275. H19/*. Ratio of humerus length to femur length (H/F): 0 = 100* H/F ≤ 65; 1 = H/F > 65, ≤ 80; 2 = H/F > 80.

Carpal bones

276. W2. Ulnar-pisiform articulation: 0 = Facet on pisiform for ulnar styloid process is roughly equal in size to that for triquetrum; 1 = Facet on pisiform for ulnar styloid process is much enlarged and deeply excavated.

Os pelvis

277. OP1/299. Gluteal tuberosity: 0 = present; 1 = absent.
278. OP2/300. Position of posterior gluteal tuberosity: 0 = Proximal to or level with lesser trochanter; 1 = distal to lesser trochanter.

Femur

279. F1*. Length of femoral neck: 0 = ≤ 75; 1 = 75-120; 2 = ≥ 120.
280. F2*. Angle of femoral neck: 0 = < 60; 1 = 60-70; 2 = > 70.

281. F3. Angle of lesser trochanter: 0 = medial (0-30°); 1 = posterior (>30°)
282. F4*. Size of third trochanter: 0 = large; 1 = small; 2 = low crest or absent.
283. F5*. Knee index (Antero-posterior diameter of distal femur/ mediolateral diameter of distal femur): 0 = < 90 (shallow knee); 1 = 90 – 100; 2 = > 100 (deep knee).
284. F6*. Femoral head shape: 0 = spherical; 1 = semicylindrical; 2 = cylindrical.
285. F7. Anterior extension of greater trochanter: 0 = no extension; 1 = extension present.
286. F8. Anterior bend of proximal femur: 0 = none; 1 = bent
287. F9*. Relative length of trochanteric fossa: 0 = long (> 125); 1 = moderate (110-125); 2 = very short (< 110).
288. F10. Presence of intertrochanteric crest: 0 = crest absent; 1 = crest present.
289. F11*. Size of lesser trochanter: 0 = large; 1 = intermediate; 2 = small.
290. F12. Lateral rim of knee: 0 = low; 1 = high.

Tibia

291. T1’. Fusion of tibia and fibula: 0 = absent; 1 = present.
292. T1”*. Articulation tibia/fibula: 0 = small; 1 = moderate; 2 = extensive.
293. T3. Shape of distal surface of tibia: 0 = square/parallel; 1 = triangular.
294. T4*. Rotation of the medial malleolus: 0 = none; 1 = slight; 2 = strong.
295. T5*. Shape of medial malleolar articular surface: 0 = flat; 1 = anteriorly convex, posteriorly flat; 2 = all convex.
296. T6. Shape of distal tibial shaft: 0 = no compression; 1 = anteroposteriorly compressed.
297. T7. Position of tibialis posterior groove: 0 = on medial side of malleolus; 1 = on posterior side of malleolus.

Talus

298. A1. Position of the flexor hallucis longus groove: 0 = lateral to trochlea; 1 = central to trochlea.
299. A2’*. Shape of talo-fibular facet: 0 = steep-sided; 1 = steep-sided with a platar lip; 2 = sloped obliquely.
300. A4’*. Development of the talar posterior trochlear shelf: 0 = none; 1 = weakly developed; 2 = well developed (prominent).
301. A5’. Talar neck length (NL/TL x 100): 0 = short (< 50); 1 = long (> 50).
302. A6. Medial talo-tibial facet: 0 = short (does not reach to plantar edge of bone); 1 = long.
303. A7/295. Lateral talar trochlear asymetry: 0 = absent; 1 = present.
304. A8/296. Talar cotylar fossa: 0 = shallow; 1 = deep, medially projecting.
305. A9’/297. Width of the head of the talus (HW/HHT x 100): 0 = < 120; 1 = > 120.
306. GEB1*. Talar neck angle: 0 = < 20°; 1 = 20-30°; 2 = > 30°.
307. GEB2*. Talar body height (HT/MTRW x 100): 0 = < 100; 1 = 100-120; 2 = 120-150.
308. GEB3*. TW/TL x 100: 0 = < 60; 1 = > 60.

Calcaneus

309. C1*. Anterior calcaneal elongation: 0 = not elongate (ACL or anterior calcaneal ratio < 40); 1 = moderate (ACL ≥ .40-45); 2 = long (> .45).
310. C2*. Position of the peroneal tubercle: 0 = distal to joint; 1 = at joint; 2 = proximal to joint.
311. C3. Posterior calcaneal bowing: 0 = absent; 1 = present.
312. C4/298. Calcaneo-cuboid articulation: 0 = articular wedge absent (fan-shaped); 1 = articular wedge present (more circular).

Navicular

313. N1*. Length relative to width: 0 = short (<90); 1 = moderate (100-150); 2 = long (>150).

314. N3. Morphology of the naviculocuboid articulation: 0 = cuboid facet on navicular contacts only the ectocuneiform; 1 = cuboid facet contacts the ectocuneiform and mesocuneiform facet.

Entocuneiform

315. E1*. Shape of entocuneiform/MT1 articulation: 0 = dorsally reduced; 1 = dorsal moiety of joint enlarged relative to ventral moiety; 2 = dorsal moiety greatly enlarged.

316. E2. Lateral process of entocuneiform: 0 = small; 1 = hypertrophied.

General Foot

317. O1. Foot axis: 0 = mesaxonic; 1 = paraxonic; 2 = ectaxonic.

318. O2. Toilet claw: 0 = absent; 1 = present.

319. O3. Prehallux: 0 = present; 1 = absent.

320. O4. Metatarsus length: 0 = short; 1 = long.

Metatarsal

321 MT1*. Peroneal tubercle of MTI: 0 = very large; 1 = large; 2 = small.

322 MT2. Hallux length: 0 = short; 1 = long.

Visual system:

323 V1/288. Optic fovea: 0 = absent; 1 = present.

324 V2/290. Tapetum lucidum: 0 = present; 1 = absent.

Character sources:

- For details about the source of most characters, see refs[7,11,12]
  - V1/288, V2/290: ref.[13]
  - C4/298, A7/295, A8/296, A9/297, OP1/299, OP2/300, H18, H19, Cr50/301, Cr51/302: ref.[14]
  - Cr52/292: ref.[15]
  - A1, A2’, A4, A5’, A6, GEB1-3: ref.[16]
  - M8-9-10’, ML18-19, ML88, ML126-127, ML147-169: ref.[17]

Supplementary References

1 Gingerich, P. D., Smith, B. H. & Rosenberg, K. R. Allometric scaling in the dentition of primates and prediction of body weight from tooth size in fossils. *Am. J. Phys. Anthropol.* **58**, 81-100 (1982).

2 Heesy, C. P. & Ross, C. F. Evolution of activity patterns and chromatic vision in primates: morphometrics, genetics and cladistics. *J. Hum. Evol.* **40**, 111-149 (2001).

3 Kay, R. F. *et al.* The anatomy of *Dolichocebus gaimanensis*, a stem platyrrhine monkey from Argentina. *J. Hum. Evol.* **54**, 323-382 (2008).

4 Marivaux, L. *et al.* Anthropoid primates from the Oligocene of Pakistan (Bugti Hills): data on early anthropoid evolution and biogeography. *Proc. Natl. Acad. Sci. USA* **102**, 8436-8441 (2005).

5 Chaimanee, Y. *et al.* Late Middle Eocene primate from Myanmar and the initial anthropoid colonization of Africa. *Proc. Natl. Acad. Sci. USA* **109**, 10293-10297, doi:doi:10.1073/pnas.1200644109 (2012).

6 Chaimanee, Y., Chavasseau, O., Lazzari, V., Euriat, A. & Jaeger, J.-J. A new Late Eocene primate from Krabi basin (Thailand) and the diversity of Paleogene anthropoids in Southeast Asia. *Proc. R. Soc. B* **280**, 20132268, doi:10.1098/rspb.2013.2268 (2013).

7 Marivaux, L. The Eosimiid and Amphipithecid primates (Anthropoidea) from the Oligocene of the Bugti Hills (Balochistan, Pakistan): New insight into early higher primate evolution in South Asia. *Palaeovertebrata* **34**, 29-109 (2006).

8 Swofford, D. L. *PAUP*. Phylogenetic Analysis Using Parsimony (*and Other Methods). Version 4*, (Sinauer Associates, 2003).

9 Seiffert, E. R. Early primate evolution in Afro-Arabia. *Evol. Anthropol.* **21**, 239-253 (2012).

10 Fulwood, E. L., Boyer, D. M. & Kay, R. F. Stem members of Platyrrhini are distinct from catarrhines in at least one derived cranial feature. *J. Hum. Evol.* **100**, 16-24 (2016).

11 Ross, C. F., Williams, B. A. & Kay, R. F. Phylogenetic analysis of anthropoid relationships. *J. Hum. Evol.*, 221-306 (1998).

12 Kay, R. F., Williams, B. A., Ross, C. F., Takai, M. & Shigehara, N. in *Anthropoid origins: new visions* (eds Callum F. Ross & Richard F. Kay) 91-135 (Springer US, 2004).

13 Martin, R. D. *Primate origins and evolution: a phylogenetic reconstruction*. (Princeton University Press, 1990).

14 Seiffert, E. R., Simons, E. L. & Simons, C. V. M. in *Anthropoid origins: new visions* (eds Callum F. Ross & Richard F. Kay) 157-181 (Springer US, 2004).

15 Beard, K. C., Qi, T., Dawson, M. R., Wang, B. & Li, C. A diverse new primate fauna from middle Eocene fissure-fillings in southeastern China. *Nature* **368**, 604-609 (1994).

16 Gebo, D. L., Dagosto, M., Beard, K. C. & Qi, T. Middle Eocene primate tarsals from China: implications for haplorhine evolution. *Am. J. Phys. Anthropol.* **116**, 83-107 (2001).

17 Marivaux, L. *et al.* A Fossil Lemur from the Oligocene of Pakistan. *Science* **294**, 587-591 (2001).
