# Supplementary material for: New Eocene primate from Myanmar shares dental characters with African Eocene crown anthropoids
Source: Nat Commun. 2019 Aug 6;10:3531. doi: 10.1038/s41467-019-11295-6 (PMC6684601; doi:10.1038/s41467-019-11295-6)
Supplement: Supplementary file 4 — Description of Additional Supplementary Files [file 41467_2019_11295_MOESM4_ESM.pdf]

Description of Additional Supplementary Files

File Name: Supplementary Data 1
Description: Datamatrix in Nexus format used for the phylogenetic analysis.
